# RNA sequencing provides novel insights into the pathogenesis of naturally occurring myxomatous mitral valve disease stage B1 in beagle dogs

**DOI:** 10.1371/journal.pone.0300813

**Published:** 2024-05-16

**Authors:** Tae-Seok Kim, Chae-Yeon Hong, Seong-Ju Oh, Yong-Ho Choe, Tae-Sung Hwang, Jaemin Kim, Sung-Lim Lee, Hakyoung Yoon, Eun-Yeong Bok, A-ra Cho, Yoon Jung Do, Eunju Kim

**Affiliations:** 1 College of Veterinary Medicine, Gyeongsang National University, Jinju, Gyeongsangnam-do, Republic of Korea; 2 Division of Applied Life Science, Gyeongsang National University, Jinju, Gyeongsangnam-do, Republic of Korea; 3 Institute of Agriculture and Life Sciences, Gyeongsang National University, Jinju, Gyeongsangnam-do, Republic of Korea; 4 Research Institute of Life Sciences, Gyeongsang National University, Jinju, Gyeongsangnam-do, Republic of Korea; 5 College of Veterinary Medicine, Jeonbuk National University, Iksan, Jeollabuk-do, Republic of Korea; 6 Division of Animal Diseases & Health, National Institute of Animal Science, Rural Development Administration, Wanju, Jeollabuk-do, Republic of Korea; The Open University, UNITED KINGDOM

## Abstract

Myxomatous mitral valve disease (MMVD) is the most common cardiovascular disorder in dogs with a high prevalence, accounting for approximately 75% of all canine heart disease cases. MMVD is a complex disease and shows variable progression from mild valve leakage to severe regurgitation, potentially leading to heart failure. However, the molecular mechanisms and age-related changes that govern disease progression, especially at the early stage (B1) before the development of discernable clinical signs, remain poorly understood. In this prospective study, we aimed to compare gene expression differences between blood samples of aged beagle dogs with stage B1 MMVD and those of healthy controls using RNA sequencing. Clinical evaluation was also conducted, which revealed minimal differences in radiographic and echocardiographic measurements despite distinct biomarker variations between the two groups. Comparative transcriptomics revealed differentially expressed genes associated with extracellular matrix remodeling, prostaglandin metabolism, immune modulation, and interferon-related pathways, which bear functional relevance for MMVD. In particular, the top 10 over- and under-expressed genes represent promising candidates for influencing pathogenic changes in MMVD stage B1. Our research findings, which include identified variations in clinical markers and gene expression, enhance our understanding of MMVD. Furthermore, they underscore the need for further research into early diagnosis and treatment strategies, as, to the best of our knowledge, no prior studies have explored the precise molecular mechanisms of stage B1 in MMVD through total RNA sequencing.

## Introduction

Myxomatous mitral valve disease (MMVD), which is characterized by gradual degeneration of the mitral valve extracellular matrix (ECM), is a pervasive cardiovascular disorder in dogs, representing approximately 75% of canine heart disease cases [1–3]. The underlying pathophysiology of MMVD is primarily attributed to myxomatous degeneration of the mitral valve and associated chordae tendineae; additionally, the tricuspid valve can also be affected by myxomatous changes [4, 5]. The degenerating heart valves become incompetent, and lead to increasing volumes of regurgitation, volume overload, associated atrial and ventricular chamber enlargement, and, in some animals, eventual congestive heart failure.

In dogs, as observed in humans, the prevalence and severity of heart disease are found to be closely age-dependent [6, 7]. Canine heart disease is typically identified during the long asymptomatic or preclinical stages and progresses slowly over the years. In dogs, with the notable exception of Cavalier King Charles Spaniels, where onset begins as early as 2 to 3 years, the prevalence of mitral regurgitation increases almost linearly with age, starting from 5 to 6 years, escalating from approximately 10% at 6 years to 60% by 12 years [4]. More than 85% of dogs over 13 years of age show evidence of valve lesions, and in most breeds, the incidence of these lesions is close to 100% by the end stage of canine life [5, 8]. Therefore, understanding the pathogenesis of canine MMVD necessitates a focus on the aging-related changes in the cardiovascular system. These changes, such as valve degeneration, increased rigidity, or even elongation and floppiness, lead to common pathologies like valve dysfunctions and diminished cardiac performance, contributing to increased morbidity and mortality in older dogs [4, 5, 8, 9].

For the specific classification of heart disease and heart failure in dogs, a consensus panel adopted a staging system that remains useful when applied to dogs with MMVD, connecting morphological changes and clinical signs to appropriate treatment at each stage [10]. According to the American College of Veterinary Internal Medicine (ACVIM) consensus classification system, MMVD stages A and B describe dogs without clinical signs, whereas stages C and D indicate those with clearly observable clinical signs of heart failure such as coughing, difficulty breathing, and exercise intolerance [5, 8]. Stage B is further divided into B1: mitral valve regurgitation with no or mild heart enlargement, and B2: advanced mitral valve regurgitation with heart enlargement. Among these MMVD stages, stage B1 is of particular interest, as it includes dogs with no radiographic or echocardiographic evidence of cardiac remodeling, as well as those showing changes that are not yet severe enough to initiate treatment, according to the ACVIM consensus guidelines for the diagnosis and treatment of myxomatous mitral valve disease in dogs [8]. This stage serves as a critical point at which the progression of the disease can be influenced and controlled; however, diagnosing this stage remains challenging without echocardiography, as patients exhibit no symptoms or minimal clinical alterations [8].

Transcriptomic profiling, coupled with bioinformatic analysis, enables the identification of gene signal intensity and gene changes between groups, along with gene ontology (GO) enrichment, network identification, functional clustering, and biological effect annotation. Such information unveils relevant signaling pathways that can then be investigated in greater detail and functionally assessed, offering powerful insights into gene functions in the disease [11]. Additionally, abundant transcriptome data reported in several studies in dogs with MMVD [12, 13] may reveal consistent changes, facilitating hypothesis-driven studies examining putative signaling pathways involved in disease pathogenesis and identifying active genes in MMVD. Therefore, further research at the transcriptomic level is necessary to achieve a more precise diagnosis and understand the intricate molecular mechanisms underlying MMVD stages. Although numerous studies have attempted to elucidate the molecular mechanisms of MMVD using various approaches such as serum proteomics, microRNA analysis, and transcriptomics [11, 14–16], data on the serum transcriptome and specific gene changes for MMVD stage B1 in dogs are limited. To the best of our knowledge, no study has investigated the specific molecular mechanisms of MMVD stage B1 using total RNA sequencing (RNA-seq), a high-throughput technique for gene expression profiling.

To address these knowledge gaps, we aimed to reveal the underlying pathogenesis of MMVD stage B1 by performing a comprehensive clinical evaluation along with the comparison of RNA-seq data to uncover gene expression disparities and potential molecular mechanisms. Our work not only enhances the understanding of age-related MMVD progression but also establishes a foundation for advanced diagnostic and therapeutic strategies to improve canine cardiovascular health.

## Materials and methods

### Study approval and enrollment

This study was approved by the Animal Care and Use Committee of the National Institute of Animal Science (NIAS 2022–586), and all procedures were conducted following relevant local and international guidelines. All dogs were housed under controlled environmental conditions and were professionally supervised at the National Institute of Animal Science (Wanju-gun, Jeollabuk-do, Republic of Korea) of the Rural Development Administration during the study. Each dog was housed in an individual room (1.7 m × 2.1 m) held at a consistent temperature (22–24°C) with consistent lighting cycles (12 h light, 12 h dark) during the experimental period. All dogs were provided approximately 3 h of outdoor activity every day with other dogs for socialization. Food was provided at an amount estimated by the maintenance energy requirement equation for each dog twice per day throughout the duration of the experiment, and drinking water was provided ad libitum. The feed or living conditions remained unaltered throughout the trial period. To prevent infectious diseases, all dogs included in the study were provided with proper care, including annual vaccination against rabies, distemper, adenovirus, parvovirus, parainfluenza, *Bordetella bronchiseptica* and canine parainfluenza virus. They were also administered regular heartworm medication (once a month) and intra-parasite preventive (every six months) as prescribed by a veterinarian. Additionally, routine health monitoring was carried out, including regular evaluation of changes in body conditions, and minimum laboratory examination like CBC and serum chemistry every 6 months.

Eight 4-year-old beagle dogs of mixed sex (neutered females *n* = 4; neutered males *n* = 4) and eight 11-year-old dogs (neutered females *n* = 8) were included in the study. The average body weight (BW) in the younger (11.05 ± 2.17 kg) and older dog (13.03 ± 3.18 kg) groups did not differ significantly. None of the dogs included in the study exhibited generalized symptoms such as decreased appetite, vomiting, diarrhea, and signs of pain, as well as specific symptoms of cardiovascular disorders such as coughing, weakness, and exercise intolerance. All dogs underwent general health monitoring, annual vaccination, regular heartworm medication administration as prescribed by a veterinarian, and comprehensive health assessments aligning with the ACVIM consensus guidelines for the diagnosis and treatment of MMVD [8], including physical examinations, hematological and serum biochemistry analysis, cardiac biomarker evaluation, and heart function assessment via thoracic radiography and echocardiography.

All 4-year-old dogs that showed no specific abnormalities during the physical, clinical, radiographic, and echocardiographic assessments were classified as healthy and placed in the normal control (NC) group (*n* = 8). The 11-year-old dogs in our study, exhibiting mitral valve regurgitation and varying degrees of left atrial and ventricular size on echocardiographic and radiographic assessments, were categorized as MMVD stage B1. These dogs had either normal or slightly enlarged left atrial (LA) and left ventricular (LV) dimensions. However, the degrees of enlargement and mitral valve regurgitation were not significant enough to meet the criteria (a murmur intensity ≥ 3/6, an echocardiographic left atrium to aorta ratio (LA:Ao) ≥ 1.6, a normalized left ventricular internal diameter at end diastole (LVIDDN) ≥ 1.7, and a vertebral heart score (VHS) > 10.5) for MMVD stage B2. Moreover, none of them met the advanced criteria for MMVD stage B2. All 11-year-old dogs except for one that did not meet the above criteria for diagnosing MMVD B1 constituted the MMVD group (*n* = 7).

### Blood sample collection

The dogs were fasted for 12 h before blood collection, and a total of 10 mL whole blood was collected from the cephalic vein using a syringe with a 21-gauge needle. The collected blood was immediately separated into ethylene diamine tetraacetate-dipotassium salt (K_2_EDTA) vacutainer tubes (BD, Franklin Lakes, NJ, USA) for measuring complete blood cell count or silicone-separator tubes (SSTs) (BD) for serum analysis. The serum was obtained from the blood in the SSTs using centrifugation (2000 × *g*, 15 min), followed by freezing and storage (−80°C) for subsequent biochemical analyses. For sequencing, a total of 3 mL whole blood was collected in Applied Biosystems Tempus Blood RNA tubes (Thermo Fisher Scientific, Waltham, MA, USA) and stored at −80°C for subsequent hematological analysis.

### Hematological and serum biochemical analyses

The complete blood count was determined using a hematology analyzer (ProCyte Dx Hematology Analyzer, IDEXX Laboratories, Westbrook, ME, USA); the serum biochemical parameters were evaluated using an automatic analyzer (Catalyst Dx Serum Chemistry Analyzer, IDEXX). Additionally, the levels of the cardiac markers N-terminal fragment of the prohormone B-type natriuretic peptide (NT-proBNP) and cardiac troponin-I (cTnI) were evaluated as biomarkers for cardiac disease in dogs using a canine NT-proBNP ELISA kit (Celltrix, Seongnam, Korea) and an Atellica IM 1600 analyzer (Siemens, Munich, Germany), respectively, according to the manufacturer’s instructions.

### Thoracic radiography and echocardiography

**Fig 1:**
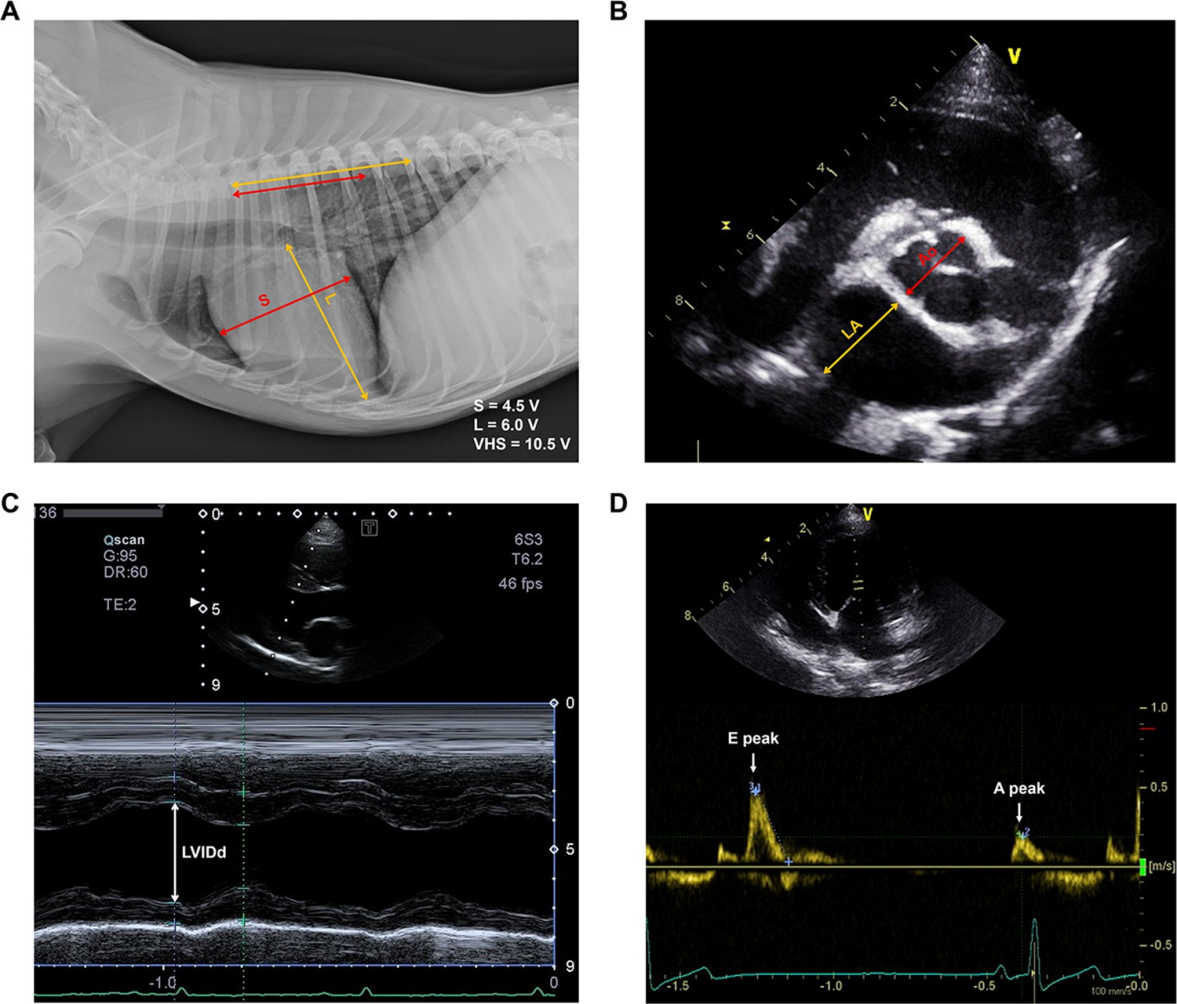
Radiological and echocardiographic imaging of the study subjects. (A) Radiographic vertebral heart score (VHS); S and L indicate the short (red) and long (yellow) axes of the heart, respectively. (B) The echocardiographic left atrium:aorta (LA:Ao) ratio was measured using the right-sided, short-axis view in the early diastole. The red arrow indicates the measurement of the aortic dimension at the level of the aortic valve; the yellow arrow indicates the measurement of the left atrial dimension. (C) The echocardiographic left ventricular internal diameter at end-diastole (LVIDd) was measured using the right-sided, long-axis view in the later diastole. The yellow arrow indicates measurements of the left ventricular internal diameter at the diastole. The ECG tracing is depicted at the bottom. (D) E-peak velocity was measured from mitral inflow profiles using the left-apical view in the early phase of ventricular filling.

Thoracic radiography and echocardiography were performed at the Animal Medical Center, College of Veterinary Medicine, Jeonbuk National University. Radiographs were obtained upon enrollment to assess cardiac size using the vertebral heart score (VHS) method as previously described (Fig 1A) [17]. Echocardiography was performed on dogs without sedation, with measurements obtained from at least three cardiac cycles; the resulting mean values were recorded. The left atrium and aorta were measured using a short-axis view at the level of the aortic valve, as previously described (Fig 1B) [18]. Additionally, the LV internal diameter at end-diastole (LVIDd) was measured using M-mode echocardiography with the right parasternal long-axis view (Fig 1C) [19]. To account for body size variations, normalized dimensions were computed using the following equation: normalized LVIDd (LVIDDN) = LVIDd (cm) / BW (kg)^0.294^ [20]. The transmittal flow velocity (E-peak), which corresponds to the initial phase of ventricular filling and spans from the opening of the mitral valve to the point of maximum ventricular filling, was also measured using the apical 4-chamber view (Fig 1D). To further evaluate diastolic function, we utilized two specific echocardiographic measurements: E/A ratio, indicative of early to late ventricular filling velocities, and E′/A′ ratio, assessing myocardial velocities during these phases using tissue Doppler imaging.

### RNA extraction, library preparation, and sequencing

RNA was extracted using TRIzol Reagent (Thermo Fisher Scientific), assessed for quality via gel electrophoresis, and quantified using the Quant-it™ RiboGreen RNA Assay Kit (Invitrogen, Carlsbad, CA, USA) according to the manufacturer’s instructions. Total RNA integrity was evaluated with a 10 nM cutoff using an Agilent 2100 Bioanalyzer (Agilent Technologies, Santa Clara, CA, USA). An Illumina TruSeq RNA Sample Prep Kit (Illumina, Inc., San Diego, CA, USA) was used to prepare RNA-seq libraries according to the manufacturer’s instructions for rRNA depletion, purification, fragmentation, and priming for cDNA synthesis. Sequencing was performed on an Illumina NovaSeq platform, producing a minimum of 2 Gb of 101-bp paired-end reads. The FASTQ files were acquired, and the data quality was assessed using Phred quality scores for each base at Q30% and Q20%. The GC content and duplication level of sequences were computed, and high-quality reads were selected for subsequent analysis. The processed reads were aligned to the *Canis familiaris* reference sequence using hierarchical indexing for spliced alignment of transcripts v2.1.0 [21]. The reference genome and sequence annotation data were downloaded from the National Center for Biotechnology Information (NCBI) Genome Assembly and RefSeq databases, respectively (https://www.ncbi.nlm.nih.gov/). The expression values were extracted using StringTie based on the reference gene model [22]. Aligned reads were assembled into transcripts, and their abundance was estimated. Expression was calculated as read counts, fragments per kilobase of exons per million fragments mapped, and transcripts per million fragments mapped per sample. To perform variant calling on RNA-seq data, the processed reads were aligned to the *Canis familiaris* genome (CanFam3.1) using STAR. Subsequently, SAMTOOLS and BCFTOOLS were employed for variant calling with the mapped reads. Each sample underwent variant filtering using the varFilter module of vcftools based on criteria including root mean square of the mapping quality (Q ≥ 20) and read depth (d ≥ 100). Statistical differences among 14,192 of the 35,993 evaluated genes were analyzed after excluding 21,801 genes that had zero counts. These profiles were used for further analyses, including the identification of differentially expressed genes (DEGs).

### DEG analysis

DESeq2 (version 1.8.1) within R software was used to compare gene expression in blood samples from dogs in the NC and MMVD groups. Preliminary quality checks were performed on the raw count data, including an assessment of non-zero counts across replicates in each group. A correlation matrix and a multidimensional scaling (MDS) plot were used to confirm the expression similarities between the samples. Log_2_-transformed (read count +1) and regularized log (rlog) transformation values were used for visualization, particularly for genes with low expression values. Statistical analysis of DEGs was conducted using the negative binomial Wald test. Volcano and log ratio vs. mean average (MA) plots were generated for visualization. Significance was determined using the nbinomLRT method, and expression differences with |fold change (FC)| ≥ 2 and a raw *p*-value < 0.05 were considered significant. Expression differences with |fold change| ≥ 2 and a raw *p*-value < 0.05 were considered significant. Hierarchical clustering was performed on the rlog-transformed values for these significant genes using Euclidean distances and the complete linkage method.

### GO and Kyoto Encyclopedia of Genes and Genomes (KEGG) analysis of DEGs

To understand the roles of the DEGs between the NC and MMVD groups, GO and KEGG analyses were conducted. Gene enrichment and functional annotation analyses were performed for significant DEGs against the GO database using the gProfiler tool [23]. Adjusted *p*-values resulting from the gProfiler output were obtained through a one-sided hypergeometric test and corrected using the Benjamini–Hochberg method. Additionally, we evaluated the data classified according to the three GO terms biological processes, cellular components, and molecular functions. KEGG pathway analyses were conducted using the KEGG database (GenomeNet, http://www.genome.ad.jp/kegg/) to gain insights into the roles and functions of genes within biological systems. Pathways with *p*-values <0.05 were recognized as significantly enriched pathways.

### Statistical analyses

Statistical comparisons between two groups of hematological and serum biochemical parameters and thoracic radiographic and echocardiographic results were performed using Prism version 8 (GraphPad, La Jolla, CA, USA). The Shapiro–Wilks test was used to check the normality of the data distribution. For datasets exhibiting normal distribution, the unpaired two-tailed *t*-test was used to evaluate statistical significance between NC and MMVD groups. Data are presented as mean ± standard deviation (SD). Statistical differences were analyzed using two-tailed unpaired *t*-test, and statistical significance was defined as *p* < 0.05.

## Results

### Hematological and biochemical parameters

The results of the complete blood count analysis revealed notable differences in certain parameters, such as red blood cell count, hematocrit levels, hemoglobin levels, and platelet (PLT) count (Table 1). The reference ranges of these parameters were determined based on the IDEXX normal reference ranges. The PLT count of dogs in the MMVD group (620.1 ± 172.2 K/μL) was approximately twice that of dogs in the NC group (317.6 ± 95.96 K/μL) (*p* < 0.001). Biochemical analysis revealed significant differences in total protein, globulin, alkaline phosphatase (ALP), and cholesterol levels between the groups. Specifically, ALP levels in the MMVD group (64.29 ± 17.90 U/L) were approximately twice those in the NC group (32.38 ± 15.25 U/L) (*p* < 0.001). The other hematological and biochemical parameters did not differ significantly between the two groups.

**Table 1:** Results of hematological and serum biochemistry analyses.

Parameter	NC (*n* = 8)	MMVD (*n* = 7)	Reference range	*p*-value
**RBC (M/μL)**	7.11 ± 0.81	5.97 ± 0.90	5.65–8.87	0.02
**HCT (%)**	45.56 ± 5.39	37.24 ± 5.70	37.3–61.7	0.01
**HGB (g/dL)**	16.01 ± 1.85	13.43 ± 1.94	13.1–20.5	0.02
**PLT (K/μL)**	317.6 ± 95.96	620.1 ± 172.2	148–484	< 0.001
**TP (g/dL)**	6.61 ± 0.18	6.68 ± 0.29	5.2–8.2	< 0.001
**GLOB (g/dL)**	3.06 ± 0.15	3.60 ± 0.48	2.5–4.5	0.02
**ALP (U/L)**	32.38 ± 15.25	64.29 ± 17.90	23–212	< 0.001
**CHOL (mg/dL)**	172.00 ± 18.49	210.1 ± 37.21	110–320	0.02

RBC, red blood cell; HCT, hematocrit; HGB, hemoglobin; PLT, platelet; TP, total protein; GLOB, globulin; ALP, alkaline phosphatase; CHOL, cholesterol. Data are presented as mean ± SD. Statistical significance was defined as *p* < 0.05 (two-tailed unpaired *t*-test).

### Thoracic radiographic and echocardiographic analyses

The outcomes for the key diagnostic indicators used for the identification of MMVD through echocardiography and radiography, along with those of cardiac biomarkers, are listed in Table 2. The reference ranges of these parameters were determined based on the findings from the thoracic radiographic and echocardiographic studies in beagles [24–26]. Significant differences were observed between the two groups in E-peak velocity (*p* < 0.001) [24], E/A ratio (*p* < 0.001) [24], and E′/A′ ratio (*p* = 0.008) [25]. Additionally, NT-proBNP (*p* = 0.03) and cTnI (*p* < 0.001) levels differed significantly between the groups. In contrast, no significant difference was observed in VHS [26], LA:Ao [24], and LVIDDN [24] values. The heart rate for the NC group (116.0 ± 10.20 bpm) and MMVD group (132.0 ± 25.46 bpm) also did not differ significantly (*p* = 0.12).

**Table 2:** Radiographic and echocardiographic assessments and levels of cardiac markers.

Parameter	NC (*n* = 8)	MMVD (*n* = 7)	Reference range		*p*-value
**VHS**	10.58 ± 0.48	10.53 ± 0.28		9.2–11.2	0.82
**LA:Ao**	1.12 ± 0.08	1.16 ± 0.17		1.0–1.5	0.65
**LVIDDN**	1.60 ± 0.14	1.50 ± 0.13		1.34–2.05	0.12
**E-peak (m/s)**	0.75 ± 0.07	0.59 ± 0.10		0.65–1.07	< 0.001
**E/A**	1.70 ± 0.33	1.12 ± 0.16		1.2–3.1	< 0.001
**E′/A′**	1.19 ± 0.34	0.76 ± 0.16		0.62–1.62	0.008
**NT-proBNP (pmol/L)**	679.90 ± 187.90	1062.00 ± 395.50		0–900	0.03
**cTnI (ng/mL)**	0.01 ± 0.004	0.02 ± 0.01		0.0–0.2	< 0.001

VHS, vertebral heart score; LA:Ao, left-atrial-to-aortic ratio; LVIDDN, normalized left ventricular internal diameter at end diastole; E/A, early to late diastolic filling velocity ratio; E′/A′, myocardial velocity ratio from tissue Doppler imaging; NT-proBNP, N-terminal fragment of the prohormone B-type natriuretic peptide; cTnI, cardiac troponin-I. Data are presented as mean ± SD. Statistical significance was defined as *p* < 0.05 (two-tailed unpaired *t-*test).

### Transcriptomic characterization of MMVD

**Fig 2:**
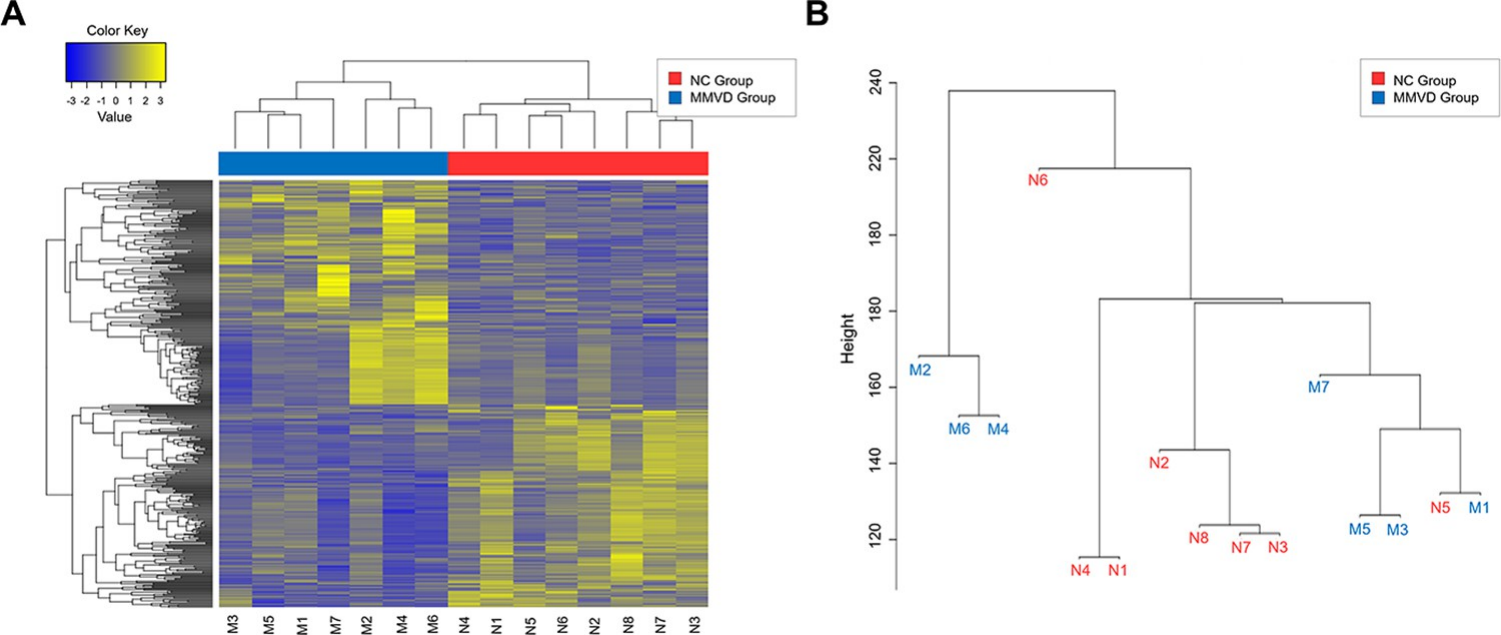
Heatmap of DEGs in beagle dogs between the NC and MMVD groups. (A) Expression of 450 DEGs in dogs in the NC (right) and MMVD groups (left). Gene expression values are based on variance-stabilizing transformed read counts for each dog. Color intensity was normalized to log_10_ (fragments per kilobase of transcript per million fragments mapped +1), with yellower colors representing more highly expressed genes and bluer colors representing less highly expressed genes. (B) Hierarchical clustering was based on the Manhattan distance among genes and is intended to highlight relative differences in expression between affected and unaffected dogs. DEG, differentially expressed gene; NC, normal control; MMVD, myxomatous mitral valve disease.

Transcriptome data for 15 dogs, including 8 NC dogs and 7 MMVD dogs, were generated from whole blood. To identify genes that showed distinct expression differences between the MMVD and NC groups, 450 DEGs were selected that met the criteria of |FC| ≥ 2 and *p* < 0.05 (Fig 2A). Hierarchical clustering analysis was performed on the significant genes to visually represent the degree of similarity in expression patterns across samples for each gene (Fig 2B).

**Fig 3:**
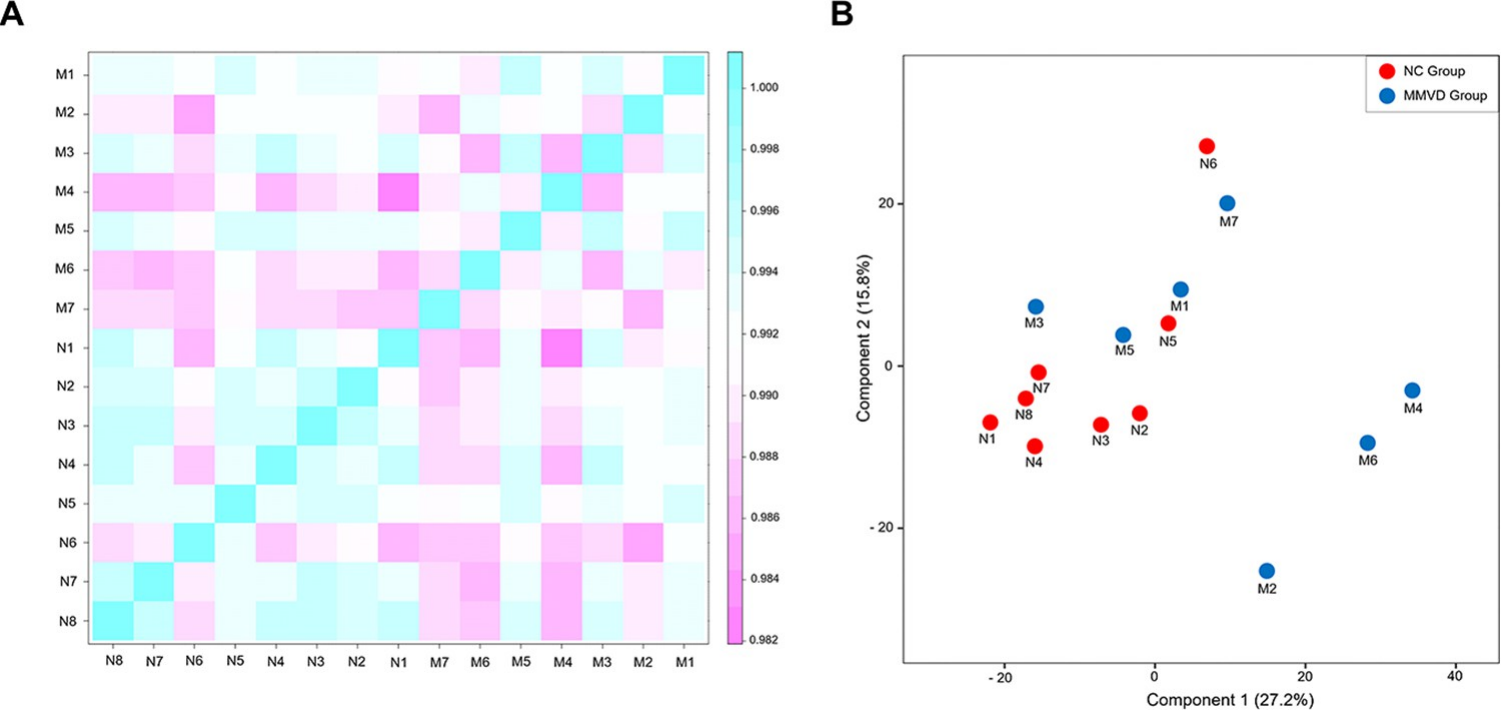
MDS plot illustrating the spatial arrangement of samples from the NC and MMVD groups. (A) Correlation matrix using normalized values for each sample, with the degree of similarity (Pearson’s correlation coefficient) between samples verifying the reproducibility of repeated samples. A coefficient value close to 1 indicates a high degree of similarity between samples. (B) MDS plot, where each data point represents an individual sample, and its placement indicates the likeness of its gene expression profile between samples. The samples from the NC and MMVD groups are depicted as red- and blue-filled circles, respectively. Some of the samples from the MMVD groups resembled to the clustered NC group samples. Samples that cluster closely together on the chart share more analogous expression patterns, whereas those that are farther apart display greater dissimilarities (i.e., M2, M4, and M6). The MDS analysis captures the inherent relationships among the samples based on transcriptomic data. MDS, multidimensional scaling; NC, normal control; MMVD, myxomatous mitral valve disease.

**Fig 4:**
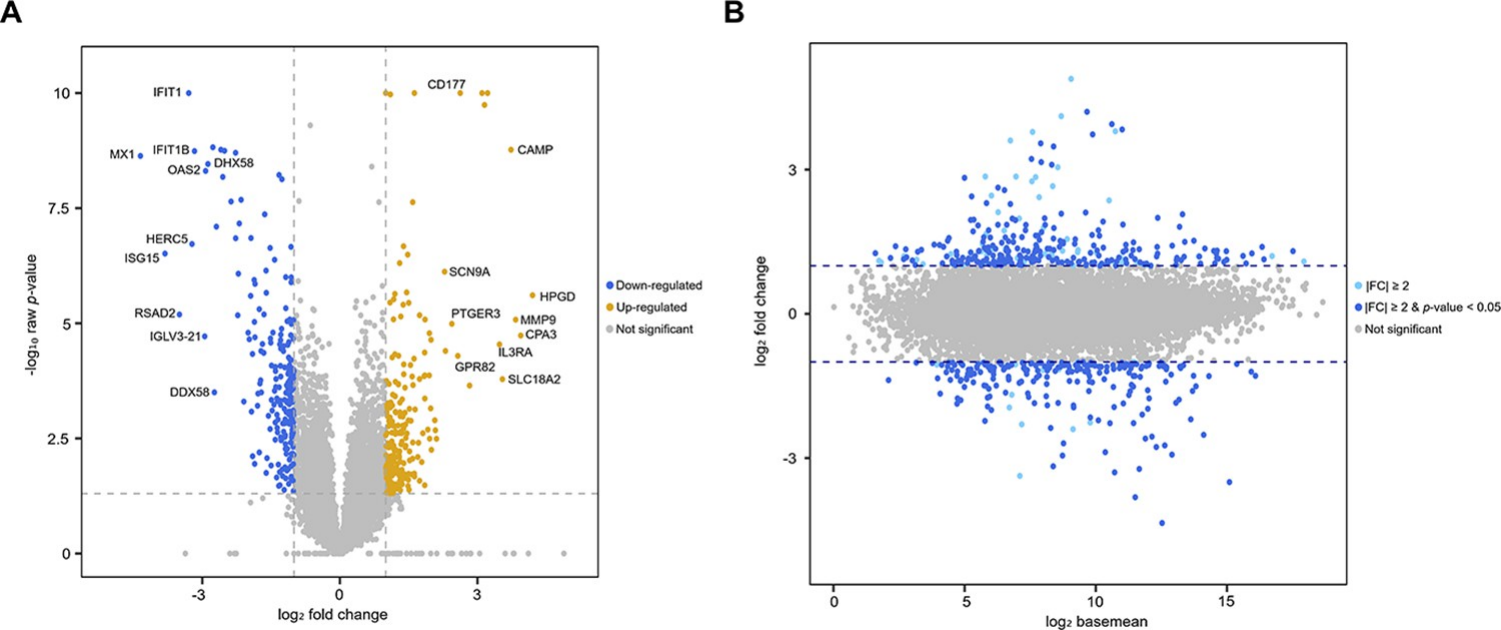
Volcano and MA plots of whole-blood RNA transcripts obtained from dogs with and without MMVD. (A) Volcano chart illustrating the association between the average log_2_ fold change (log_2_FC) and the negative base-10 logarithm of the raw *p*-values for genes that exhibit significant differential expression between groups (DEGs). Within this graph, orange dots signify transcripts (*n* = 236) displaying marked upregulation (FC ≥ 2, *p* < 0.05) between the NC (*n* = 8) and MMVD (*n* = 7) groups. Similarly, blue dots denote transcripts demonstrating significant downregulation (FC ≤ −2, *p* < 0.05; *n* = 214). In contrast, gray dots represent transcripts where the disparities in expression between groups lack statistical significance. (B) Presented in an MA plot, the gene expression data are depicted as a scatterplot on a two-dimensional plane, showing the log₂ ratio of expression values across two time points. In MA plots, the y-axis represents the log₂ FC, whereas the x-axis portrays the log₂ base mean. Each dot corresponds to an individual gene. Blue dots indicate transcripts with large, significant expression changes (|FC| ≥ 2 and *p* < 0.05) between the NC (*n* = 8) and MMVD (*n* = 7) groups; light blue dots indicate transcripts with large intergroup expression changes (|FC| ≥ 2), regardless of statistical significance. MA, log ratio vs. mean average; NC, normal control; MMVD, myxomatous mitral valve disease.

Additionally, the genetic relationships between the NC and MMVD groups were captured in a correlation matrix (Fig 3A) and an MDS plot (Fig 3B). Notably, the MDS analysis demonstrated that components 1 and 2 explained 27.2% and 15.8% of the total variance, respectively. Moreover, the differences in gene expression between the MMVD and NC groups were illustrated using a volcano plot (Fig 4A) and an MA plot (Fig 4B). These plots highlight 10 significantly upregulated genes (*HPGD*, *CPA3*, *MMP9*, *CAMP*, *SLC18A2*, *IL3RA*, *CD177*, *GPR82*, *PTGER3*, and *SCN9A*) and 10 significantly downregulated genes (*MX1*, *ISG15*, *RSAD2*, *IFIT1*, *HERC5*, *IFIT1B*, *IGLV3-21*, *OAS2*, *DHX58*, and *DDX58*) in the MMVD group compared with those in the NC group (Table 3).

**Table 3:** Top 10 over- and under-expressed genes in beagle dogs from the MMVD group vs. NC group.

Gene symbol	Fold change^a^	*p*-value	Gene name
*HPGD*	18.40	0.000619038	15-Hydroxyprostaglandin dehydrogenase
*CPA3*	15.40	0.002383047	Carboxypeptidase A3
*MMP9*	14.25	0.001382953	Matrix metallopeptidase 9
*CAMP*	13.28	1.76611×10^−6^	Cathelicidin antimicrobial peptide
*SLC18A2*	11.67	0.009653375	Solute carrier family 18 member A2, transcript variant X2
*IL3RA*	11.16	0.00302417	Interleukin 3 receptor subunit alpha, transcript variant X2
*CD177*	8.58	2.01832×10^−8^	CD177 molecule
*GPR82*	5.95	0.004557741	G protein-coupled receptor 82
*PTGER3*	5.43	0.001554784	Prostaglandin E receptor 3
*SCN9A*	4.93	0.003867723	Sodium voltage-gated channel alpha subunit 9, transcript variant X5
*MX1*	−20.39	1.96578×10^−6^	MX dynamin like GTPase 1
*ISG15*	−14.06	0.000112381	ISG15 ubiquitin-like modifier
*RSAD2*	−11.30	0.001224749	Radical S-adenosyl methionine domain containing 2
*IFIT1*	−9.82	7.68421×10^−8^	Interferon-induced protein with tetratricopeptide repeats 1
*HERC5*	−9.35	7.80231×10^−5^	HECT and RLD domain containing E3 ubiquitin protein ligase 5
*IFIT1B*	−9.0	1.76611×10^−6^	Interferon-induced protein with tetratricopeptide repeats 1B
*IGLV3-21*	−7.71	0.002407021	Immunoglobulin lambda variable 3-21-like
*OAS2*	−7.61	3.50754×10^−6^	2’-5’-Oligoadenylate synthetase 2
*DHX58*	−7.35	2.77325×10^−6^	DExH-box helicase 58, transcript variant X3
*DDX58*	−6.82	1.76611×10^−6^	DexD/H-box helicase 58

NC, normal control; MMVD, myxomatous mitral valve disease.

^a^Fold change is defined as a change in expression between the NC and MMVD groups.

### Enriched pathways of DEGs

**Fig 5:**
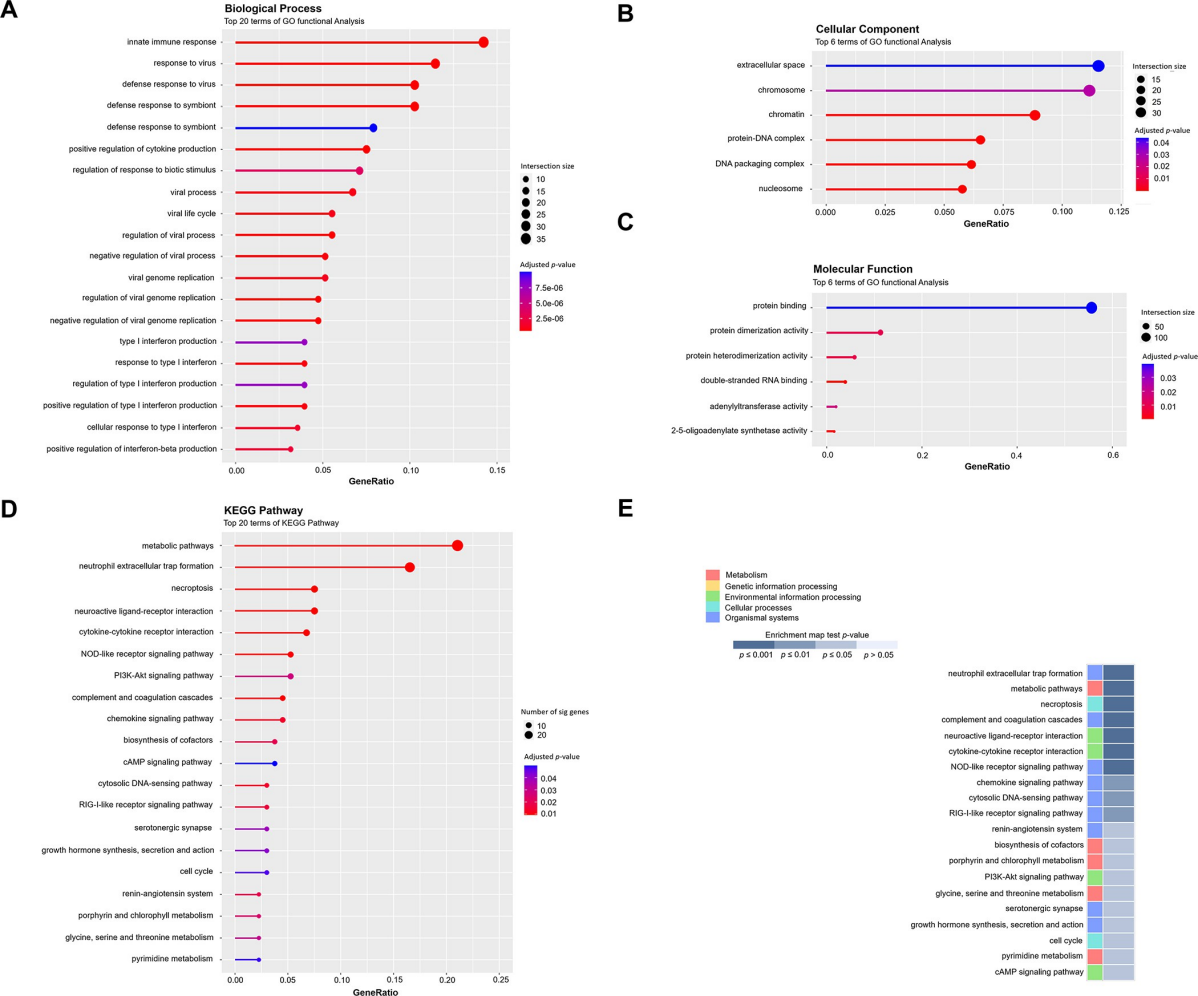
GO enrichment analysis of differentially expressed genes. (A) The top 20 terms concerning biological processes; (B) the top 6 terms corresponding to molecular functions; and (C) the top 6 terms linked to cellular components are presented in dot-and-bar graphs. The color of the dots transitions from blue to red based on the adjusted *p*-value, with dot size being proportionate to the gene count. (D) KEGG pathway annotation for genes with differential expression is shown. The dot plot showcases the 20 most significantly enriched KEGG pathways in terms of functionality. Each circle symbolizes the correlation between the gene and the pathway, with larger circles signifying a stronger relationship. The bar plot illustrates the top 20 significantly enriched functional KEGG pathways (*p* < 0.05). The enrichment score was computed based on the number of annotated genes within the pathway term; for further details, refer to the Materials and Methods section. A lower *p*-value indicates a more pronounced enrichment of the pathway. (E) The five main classifications (metabolism, genetic information processing, environmental information processing, cellular processes, and organismal systems) and the *p*-value of each KEGG pathway enrichment are shown in the heatmap of KEGG enrichment analysis. GO, gene ontology; KEGG, Kyoto Encyclopedia of Genes and Genomes.

GO analysis was employed to identify the DEGs and display the relevant biological process, cellular component, and molecular function terms linked to the target genes. Statistical significance for the GO analysis data was set at adjusted *p* < 0.05. The DEGs exhibited enrichment across 3,866 terms in the biological process category, 410 terms in the cellular component category, and 683 terms in the molecular function category. Fig 5 illustrates the most enriched GO terms for the DEGs, focusing on the top 20 terms in biological processes (Fig 5A), the leading 6 terms in cellular components (Fig 5B), and the top 6 terms in molecular functions (Fig 5C).

The top 20 biological processes revealed heightened expression of biological functions linked to “viral infections,” “virus-related responses,” “immunity,” and “innate immunity.” Cellular component analysis highlighted the differential expression of transcripts associated with the regulation of gene expression, including terms such as “chromatin,” “protein–DNA complexes,” “DNA packaging complexes,” and “nucleosomes.” In terms of molecular functions, DEGs were notably enriched in functions like “protein binding,” “protein dimerization activity,” “protein hetero-dimerization activity,” “double-stranded RNA binding,” “adenylyl-transferase activity,” and “2-5-oligoadenylate synthetase activity.” Through KEGG functional annotation, 363 pathways were categorized based on statistical significance set at *p* < 0.05. The top 20 KEGG pathways were identified as enriched with DEGs based on the gene count (Fig 5D and 5E). In the KEGG pathway analysis, transcripts exhibiting significant expression differences between the NC and MMVD groups displayed heightened enrichment of pathways associated with the “metabolic pathway.” Moreover, immune- and endocrine-related pathways showed high enrichment levels.

## Discussion

In this prospective study, we aimed to elucidate the differences in gene expression in the blood of MMVD stage B1 and NC beagle dogs using RNA-seq. This approach detects subtle changes in gene expression that cannot be easily observed using traditional methods. Furthermore, integrating clinical data with transcriptomic analysis provides a more comprehensive understanding of the disease phenotypes. This understanding will assist in predicting the direction of disease progression and the prognosis for MMVD in the future, as well as in identifying treatment strategies. In this process, beagle dogs were chosen as an ideal model for exploring naturally occurring MMVD because they were consistently raised in the same environment, allowing maximization of control over environmental variables excluding underlying diseases. This study provides credible identification of genetic changes associated with age-related cardiac function shifts and MMVD development excluding environmental impact.

In accordance with the ACVIM consensus, which classifies MMVD stage B1 as asymptomatic dogs with mitral valve regurgitation and no or mild heart enlargement [8], our results indicated no significant differences between the NC and MMVD groups in the indices reflecting changes in heart size, such as VHS, LA:Ao, and LVIDDN. However, the MMVD group exhibited a notable decrease in E-peak velocity, E/A ratio, and E′/A′ ratio compared with the NC group, indicating potential cardiac diastolic function changes.

Although advanced age is known to be associated with a decline in cardiac diastolic function and affects the echocardiographic parameters, including E-peak velocity, E/A ratio, and E′/A′ ratio [27], the significance of these changes in the context of MMVD is complicated. In MMVD, these parameters decrease initially in early stages, like stage B1, but potentially increase in later stages, such as stages C and D [28, 29]. This pattern suggests that the observed alterations in our study are more directly related to MMVD progression rather than being solely attributed to age, indicating a complex interaction between age and MMVD in influencing diastolic function in dogs.

To evaluate the extent of atrial and ventricular wall stress in dogs included in our study, we assessed the expression levels of two biomarkers, NT-proBNP and cTnI, in conjunction with thoracic radiography and echocardiography. NT-proBNP, which is released by the cardiac tissue in response to increased stress on the atrial and ventricular walls [30], is a potential predictor of heart failure and overall survival in dogs with MMVD of various stages [31, 32]. Our results revealed higher levels of NT-proBNP in the MMVD group than that in the NC group, possibly due to MMVD progression. The increase suggests a mild cardiac impact, likely related to MMVD stage B1, which involves subtle cardiac changes affecting heart function. Similarly, the level of cTnI was elevated in the MMVD group. cTnI reflects continuous myocardial injury resulting from cardiac remodeling in MMVD, with its concentration increasing as the disease progresses [33]. While increases in cTnI levels have been associated with MMVD severity and age-related cardiomyopathy [34–36], our study demonstrated that cTnI levels in both MMVD and NC groups were within the normal range. Therefore, correlating the clinical significance of the level variations of these markers, particularly in MMVD stage B1, with specific disease processes is challenging.

Among the parameters evaluated during the hematological and biochemical examinations performed as a part of the clinical evaluation, PLT counts and ALP levels displayed particularly notable variations between the MMVD and NC groups. Elevated PLT counts and ALP levels in the MMVD group compared with those in the NC group could be potential indicators of underlying physiological changes. Altered PLT function could potentially contribute to the development of MMVD owing to turbulent and high-velocity flow, along with modifications in the fluid shear stress surrounding mitral valve prolapse [37]. Similarly, increased levels of ALP could reflect increased metabolic activity or cellular turnover associated with disease progression [38, 39]. However, there are limitations in understanding the definitive mechanisms driving these elevated levels and their specific implications in MMVD.

To gain evidence-based insights regarding the potential functions of the identified DEGs in the initiation and progression of age-related MMVD, we further evaluated the top 10 over- and under-expressed DEGs using transcriptomic data. Among these DEGs, some were directly or indirectly related to mechanisms presumed to be involved in MMVD pathogenesis and to processes such as ECM remodeling and tissue degeneration, prostaglandin metabolism and signaling, inflammatory responses and immune regulation, and interferon (IFN)-related pathways, suggesting their potential as promising candidates for influencing pathogenic changes in MMVD stage B1. In particular, the enrichment of the overexpressed DEG *MMP9* in ECM remodeling and tissue degeneration highlights the likely role of ECM remodeling in age-related MMVD. *MMP9* encodes a matrix metalloproteinase that degrades ECM components [40]. Its increased expression could lead to an imbalance in matrix homeostasis, thereby weakening cardiac tissues and disrupting valve integrity. This, in turn, may enhance the progression of MMVD by rendering the heart structures more susceptible to degeneration and regurgitation. Notably, human studies have confirmed increased *MMP9* expression in myxomatous heart valves relative to that in normal valves [41, 42]. Similarly, microarray analysis showed a 10-fold increase and quantitative polymerase chain reaction analysis showed a 100-fold increase in *MMP9* expression in MMVD versus NC dogs [43, 44]. Our RNA-seq results also demonstrated a 14-fold increase in *MMP9* expression in the MMVD group compared with that in the NC group. Together, these findings highlight *MMP9* as a robust biomarker candidate for the diagnosis of MMVD stage B1 in dogs.

Additionally, we identified two genes (*HPGD* and *PTGER3*) related to prostaglandin metabolism and receptors that were overexpressed in the MMVD group. The roles of prostaglandins are influenced by enzymes involved in their metabolic pathways, together with genetic variations that affect their efficacy. Prostaglandins constitute a group of ubiquitous, naturally occurring substances that exhibit a notably broad range of biological effects, particularly within the cardiovascular system. In this context, they play pivotal roles in the regulation of vascular constriction, cardiac restructuring, and inflammatory responses [45, 46]. *HPGD* encodes 15-PGDH, an enzyme that regulates prostaglandin E2 (PGE2) metabolism and is crucial for inflammation control. 15-PGDH malfunction promotes cardiac fibrosis in infarcted hearts by degrading PGE2, leading to increased collagen levels due to transforming growth factor beta 1 activation [47]. *PTGER3* encodes EP3, the PGE2 receptor, and was identified as a candidate gene for blood pressure regulation in human studies [48]. The overexpression of the EP3 receptors in mice resulted in marked myocardial hypertrophy [49]. Although the contribution of these DEGs to cardiovascular disease pathogenesis is mainly documented in humans, elevated *HPGD* and *PTGER3* expression in dogs may amplify the effects of prostaglandins on these processes, potentially contributing to the inflammatory environment and vasodilation associated with MMVD.

We found that most of the overexpressed genes in the MMVD group, including *CPA3*, *CAMP*, *SLC18A2*, *IL3RA*, *CD177*, *GPR82*, and *SCN9A*, were associated with immune and inflammatory responses, with variable degrees of relevance to the pathogenesis of age-related MMVD in dogs. Among these, *CPA3*, *CAMP*, and *IL3RA* have been found to exhibit strong connections with cardiovascular disorders. Altered expression of CPA3, a zinc metalloprotease encoded by the *CPA3* gene and released from mast cells, has crucial implications for cardiovascular conditions, musculoskeletal disorders, and immunogenesis [50]. Elevated *CPA3* expression in age-related MMVD suggests heightened mast-cell-driven immune responses, potentially intensifying the inflammatory milieu and accelerating degenerative alterations. CAMP exhibits a complex role, as it can trigger platelet activation and blood clot formation in mice [51] while also providing protection against cardiac fibrosis in diabetic mouse hearts, showing promise as a therapeutic target for heart failure [52, 53]. The overexpression of *CAMP* observed in MMVD thus necessitates comprehensive research to unravel its precise role in the disease, given its intricate interactions with immune responses, inflammation, clot formation, and cardiac fibrosis. Additionally, *IL3RA* overexpression in MMVD indicates heightened immune cell responsiveness within the valve tissues, which is important in the context of immune-related aspects of the disease and its association with cardiovascular conditions through the JAK/STAT signaling pathway [54, 55]. However, it is important to acknowledge that the precise mechanisms through which these genes influence age-related MMVD in dogs require further investigation to establish their definitive roles in the pathogenesis of the disease.

In contrast, we found that most of the top 10 under-expressed genes, *MX1*, *ISG15*, *RSAD2*, *IFIT1*, *HERC5*, *IFIT1B*, *IGLV3*-*21*, *OAS2*, *DHX58*, and *DDX58*, were related to potential antiviral functions. Under-expression of *MX1*, *ISG15*, *RSAD2*, *IFIT1*, and *OAS2* may weaken the ability of the immune system to effectively counter viral infections, leaving the heart tissues susceptible to viral invasion and potentially contributing to MMVD progression [56]. For example, diminished protein expression of MX1, renowned for its antiviral activity, might compromise the host’s ability to curb viral replication, while reduced levels of ISG15 may dampen antiviral immunity, granting viral agents a greater influence over cardiac tissues. Multiple studies have elucidated the complex relationship between IFN-related genes and cardiovascular disorders. In one study, *ISG15*, *IFIT3*, *OAS3*, and *RSAD2* were prominently enriched in the type I IFN signaling pathway, serving as central hub genes associated with myocarditis-related immune processes, suggesting their potential involvement in the mechanism of myocardial injury in dermatomyositis [57]. Another study screened key genes linked to mitochondrial function in heart failure, including the IFN-related genes *RSAD2* and *MX1*, which exhibited substantial enrichment in myogenesis and hypoxia processes [58]. Furthermore, *ISG15*, which encodes a ubiquitin-like protein with widespread expression across vertebrate cell types, plays a pivotal role in suppressing viral replication and mitigating infectious agents within human cardiomyocytes, thereby reducing the occurrence of inflammatory cardiomyopathy, heart failure, and mortality [59]. Notably, *ISG15* has been recently linked to multiple mechanisms associated with aging and age-related cardiovascular disorders, encompassing heightened genomic DNA damage, telomere shortening, hypertension, type II diabetes, and obesity [60]. Given its significant effect on age-related cardiovascular diseases in humans, its potential association with age-related MMVD in dogs appears promising. Although the under-expression of these antiviral-related genes suggests a possible link to MMVD progression by weakening the ability of the immune system to counter viral infections in heart tissues, further research is needed to establish these genes as meaningful biomarkers for MMVD. Understanding the roles of these genes in the context of MMVD could provide valuable insights into the functional mechanisms of the disease. The potential role of the under-expressed genes involved in antiviral functions is reinforced by the results of GO analysis, which highlighted the processes related to viruses and type I IFN. Type I IFNs play a central role in immune responses against viral and microbial infections [61, 62]. Viruses can directly damage the heart by infecting and rupturing host cells and can cause indirect damage by stimulating pro-inflammatory cytokine production and attracting immune cells [63, 64]. Most genes linked to antiviral processes showed higher expression in the NC group than in the MMVD group, suggesting that the MMVD group may be more susceptible to viruses, potentially leading to an inflammatory environment in the heart [65]. Moreover, in our study, most genes related to type I IFN showed higher expression in the NC group than in the MMVD group, indicating a potential interaction between viral infection and immune response, consistent with the essential role of type I IFNs in the defense against viral infections.

In the molecular function analysis, the NC group showed increased expression of genes associated with protein binding, particularly *LRIG3*. The primary function of this gene is to activate latent ribonuclease L, which effectively suppresses infections. Leucine-rich repeat proteins, such as LRIG3, interact with various molecules and contribute to the innate immune response [66, 67]. The MMVD group appeared to be less successful in regulating initial inflammation because of the lower expression of pre-inflammatory phase transcripts than that of the NC group, and the NC group may have had a stronger and quicker response to early inflammatory triggers. Additionally, this analysis highlighted a genetic correlation with antiviral activity, showing increased levels of 2′-5′-oligoadenylate synthetase in the NC group. This enzyme plays a crucial role in suppressing viral replication and provides robust defense against viral infections [68].

KEGG pathway analysis revealed significant differences in gene expression between the NC and MMVD groups, emphasizing the importance of metabolic pathways. Dysfunctional metabolic pathways, when altered pathologically, lead to impaired signal transmission and disruptions in energy and redox balance, ultimately causing contractile dysfunction in the diseased heart [69]. This indicates the existence of notable changes in metabolic activity between the MMVD and NC groups. In the MMVD group, a total of 20 genes, including *CA6*, *PMM1*, and *NDUFS3*, were overexpressed (FC > 2) relative to levels in the NC group. Comparing these metabolic pathways with those in other disease groups or healthy controls can offer unique insights regarding the specific metabolic changes that occur in patients with MMVD [70]. Moreover, the older age of the MMVD group, compared to that of the NC group could also have contributed to age-related metabolic decline. Together, these findings reveal the complex interplay between metabolism, immune responses, inflammation, and viral defense mechanisms, enhancing our understanding of MMVD development.

Another crucial aspect to consider is the interpretation of the MDS plot obtained in this study. The MDS plot unveiled two distinct clusters within the MMVD group, one of which closely resembled the NC group. While individuals in this cluster may remain asymptomatic like those in the NC group, some may progress to advanced stages of MMVD; therefore, discerning significant differences within the stage B1 group posed a challenge. This difficulty stemmed primarily from a relatively small sample size, which made it challenging to investigate this transition in the current context thoroughly. Additionally, the exploration of demographic factors such as age and breed and their potential influence on gene expression patterns requires further investigation. Examining these variables could further elucidate the observed variability and enhance our understanding of MMVD dynamics.

In conclusion, this study employed RNA-seq to investigate gene expression disparities between aged beagle dogs with MMVD stage B1 and healthy NC subjects, focusing on transcripts that are strongly associated with age and induce significant functional changes in MMVD-related gene expression. Although separating age-related changes from those specifically linked to MMVD is challenging, our analysis emphasizes alterations more likely attributable to the disease process rather than just aging.

Our findings not only shed light on the potential molecular pathways driving the disease but also uncover subtle clinical distinctions. The changes detected in cardiac markers and the identification of genes linked to ECM remodeling, prostaglandin metabolism, immune modulation, and IFN pathways enhance our understanding of age-related MMVD. The use of genetic criteria along with clinical diagnostic standards can significantly enhance the reliability of MMVD stage B1 classification, which seems to be more sensitive in predicting disease progression compared to other advanced stages. Nevertheless, to explore its potency as a valuable tool for evaluating disease prognosis, the inclusion of additional indicators is essential to address the potential variability in the clinical diagnosis of stage B1. The findings of this study not only provide valuable insights for future mechanistic studies but also offer a clinical means to more accurately identify MMVD stage B1 in dogs.
